# The changes that occur in the immune system during immune activation in pre-diabetic patients of all ethnicities, from the age of 25- to 45-years: A systematic review and meta-analysis

**DOI:** 10.1097/MD.0000000000030903

**Published:** 2022-12-23

**Authors:** Nomusa Christina Mzimela, Aubrey Mbulelo Sosibo, Phikelelani Siphosethu Ngubane, Andile Khathi

**Affiliations:** a School of Laboratory Medicine and Medical Science, College of Health Sciences, University of Kwa-Zulu Natal, Durban, South Africa; b Department of Human Physiology, School of Laboratory Medicine and Medical Sciences, College of Health Sciences, University of KwaZulu-Natal, Durban, South Africa.

**Keywords:** immune cells, inflammatory markers, meta-analysis, pre-diabetes, systematic review

## Abstract

Pre-diabetes is an intermediate state between normoglycaemia and type 2 diabetes (T2D). This condition has been shown to be asymptomatic thus making it hard to investigate the changes that occur in the body during this state. Recent findings stipulate that in this state, there are changes that are often associated with T2D. These include changes in concentration of immune cells and inflammatory markers. This systematic review will provide a synthesis of the data that is available reporting on the changes in the concentration of immune cells and selected markers during prediabetes. It will also give clarity of the variation of the complications of the condition among the various demographic groups.

**Methods:**

The assembly of this systematic review was through strict adherance to the PRISMA 2020 guidelines for reporting systematic reviews. This systematic review has been registered with the International Prospective Registry of Systematic Reviews (PROSPERO), registration number “CRD42020184828” dated 05-07-2020). In this systematic review, published clinical studies articles that involve observational reports, whether it is case-control, cross-sectional, and comparative cross-sectional will be used. Cohort study designs that involve normal/non-diabetic and pre-diabetes reports will be used in this systematic review and meta-analysis. Clinical MeSH headings to search on MEDLINE, COCHRANE library, EMBASE, and ICTRP and African Journal Online will be a tool used to achieve the required report. Reviewers (NCM, AMS, and AK) will screen all the results and select the studies that will be eligible by guidance according to eligibility criteria. Downs and Black Checklist will be used to check the risk of bias and then for meta-analysis Review Manager v5.4 Forrest plot will be used. Additionally, the Forrest plot will also be used for sensitivity analysis. The strength of evidence will then be assessed using the Grading of Recommendations Assessment, Development, and Evaluation approach.

**Results:**

Only 4 reports were eligible and risk of bias checked. The results indicated the outcomes even though there were only few reports.

**Discussion and conclusion:**

This systematic review will give an indication on the available data on this research area and lay a foundation for future studies.

## 1. Introduction

Pre-diabetes is an intermediate state between normoglycemia and the onset of type 2 diabetes (T2D) stage.^[1]^ This condition is characterized by blood glucose concentrations being higher than normal but below the threshold for diagnosis of T2D.^[2,3]^ According to projections made by the International Diabetes Federation in 2019, the number of diabetic adults (20–79 years) in Africa is 19 million, where South Africa has the highest with 4.6 million diabetic adults.^[3]^ In 2017, the Indian population within South Africa had been reported to have the highest prevalence (11–13%) of diabetes in the country, followed by the Coloured population with 8% to 10%, then Blacks with 5% to 8% and Whites being lowest with 4%.^[4,5]^ The Indian population has been shown to be high due to their strong genetic predisposition to developing T2DM.^[4,5]^ The statistics from the International Diabetes Federation indicate that South Africa has a high prevalence of T2D and also postulate that there is also a high prevalence of undiagnosed pre-diabetes.^[5]^ The pre-diabetic state is often asymptomatic, thus making it difficult to document the prevalence of the condition. Recently, this condition has been the focus of many studies so as to identify and understand the metabolic and signaling complications occurring in this condition. The eThekwini district is an ethnically and culturally diverse area which is South Africa’s third-largest city. Additionally, it offers a convenient lifestyle with a host of amenities that cater to every budget enabling easy access to consumption of high calorie diets and sedentary lifestyles. These living conditions raise the risk of developing metabolic disorders such as pre-diabetes and T2D. Taking into account these statistics, it is then of utmost importance to report on the abnormalities that occur during pre-diabetes. More recently, studies in our laboratory using animal models have shown that complications often associated with T2D begin during the pre-diabetic state.^[6–11]^ In these studies, it was shown that the presence of pre-diabetes could lead to compromised immunity.^[6,7]^ These reports indicated that during the progression from pre-diabetes to overt T2D, there are changes in immune cell concentrations (neutrophils, lymphocytes, monocytes, eosinophils, basophils) and upregulation of inflammatory markers (C-reactive protein [CRP], tumour necrosis factor-alpha [TNF-α], Interleukin-6 [IL-6], P-Selectin, cluster of differentiation 40 ligands [CD40L], and Fibrinogen).^[7]^ This report on animal models lays a platform for research on individuals that are found to be pre-diabetic. Globally, there are reports on innate immunity based on immune cell changes and inflammation during pre-diabetes. One of the earliest research on one of the immune cells was done by Hitchcock and his coworkers on lymphocyte subsets during pre-diabetes in 1986.^[12]^ Hitchcock and his coworkers only used 65 participants and the focus was only on lymphocytes.^[12]^ However, based on the obtained data, it shows that the researcher’s focus was not on pre-diabetes and immunity until the increase in statistics of T2D around the 1990s and 2000s.^[13–16]^ Research on pre-diabetes in South Africa or specifically in the eThekwini district is very scarce even though the rate of the increase in T2D is very high. Additionally, there is no data obtained based on pre-diabetes and immune activation on different ethnicities at the eThekwini district. The average age of diagnosis for T2D in South Africa is between 55- to 65-years old while prediabetes is said to last between 10 to 20 years before the onset of T2D.^[17]^ Even among the age of 25- to 45-years, no data is reporting on immune cells and selective markers on pre-diabetic human subjects in the eThekwini district. This systematic review sought to identify the gaps regarding changes in the immune system during pre-diabetes. Another objective was to highlight differences amongst the different genders and ethnicities of people living in the eThekwini District.

## 2. Methods

Adhering to the preferred reporting items for systemic reviews and meta-analysis (PRISMA) 2015 guidelines for reporting protocols (PRISMA checklist attached in additional file 1, http://links.lww.com/MD/H558), the protocol was registered with the International Prospective Registry of Systematic Reviews (PROSPERO) registration number “CRD42020184828” dated 05-07-2020).

### 2.1. Eligibility criteria for the study

Only studies with a minimum of 100 population size and community-based clinical cross-sectional studies were eligible. This study worked with stored blood samples. The inclusion and exclusion criteria were as follows.

*Inclusion*: The information reported from non-diabetic adults within the ages of 25-45 of all ethnicities was used.

*Exclusion*: Information reported from people with a history of liver disease, kidney disease, heart disease and depression were not used. Information from pregnant women was also not used. Additionally, no studies from professional sports athletes were allowed in the study. The full-text article/reports that indicate that the subjects that were used were free from all the mentioned criteria were then eligible.

### 2.2. Ethics approval and consent to participate

The data that will be analyzed will be the data that is published and there will be no data collection from subjects. The authors declare that there will be no informed consent required to be signed and therefore no ethics approval required for the systematic review and meta-analysis

### 2.3. Pre-diabetes diagnosis criteria

Diagnostic criteria for pre‐diabetes were as follows (participants should meet one of the following diagnoses): fasting blood glucose: 5.6 to 7.0 mmol/L; 2 hours postprandial blood glucose (2 h–OGTT): 7.8 to 11.0 mmol/L with glycated hemoglobin (HbA1c): 5.7% to 6.4%.

### 2.4. Information sources

The information sources were any reported clinical study that involved a minimum of 100 participants, either males or females and both genders, aged from 25 to 45 years from all ethnicities. These clinical studies involved observational studies if they were cross-sectional, comparative cross-sectional, case-control or cohort study designs that involve normal and pre-diabetes reports. The reports that contained information that involves specifically one or more immune cells (neutrophils, lymphocytes, monocytes, eosinophils, basophils) in the pre-diabetic state were eligible. Additionally, any study that reports information that involves at least one or more inflammatory markers of interest, which are CRP, TNF-α, IL-6, P-Selectin, CD40L, and fibrinogen was also eligible.

### 2.5. Search strategy

The electronic search strategy was used for identification of studies involving cohorts that have been done that are related to the study of interest. This strategy was accomplished by search on MEDLINE (from 1963 to 2020), COCHRANE library displaying results of trials from PubMed, CT.gov, EMBASE, and ICTRP (from 1963 to 2020) and African Journal Online (from 1998 to 2020). Added to these search strategies, the use of clinical MeSH headings and text words was applied to filter the available information. For all searches done, the keywords used were “pre-diabetes and immunity,” “pre-diabetes and immune cells,” “pre-diabetes and leucocytes,” and “pre-diabetes and inflammation.”

### 2.6. Identification of eligible studies

The title and abstracts of all the obtained results were screened by reviewers (NCM, AMS, and AK) and the studies that met the eligibility criteria were then selected. Basically, each reviewer was responsible for screening all the selected study reports before the decision making of the eligible reports. The PRISMA flow chart for the selection of studies is shown in Additional file 2, http://links.lww.com/MD/H559. The author was contacted twice if data reported was unclear for clarity.

### 2.7. Study records and data extraction

The data of study records selected as eligible reports were then extracted and recorded in a Microsoft Excel file. The pre-defined list of variables to be considered in each and every report was used as categories in a Microsoft Excel file. Considering the research of interest, the outcome of interest was mainly the immune cell response and concentration of selective markers in both genders, at an age parameter of interest in all ethnicities. However, the value of the baseline characteristic of the data reported was considered. Therefore, the baseline characteristics of eligible research reports obtained were author, year of publication, country and study setting. The methodology of the study reported was then considered with the categories (design, time period, sampling strategy and whether participants are normal or pre-diabetic population) considered. Finally, the outcomes from different gender, ages, ethnicity, immune cell changes/inflammatory markers were then extracted.

#### 2.7.1. Data simplification.

Studies that report on the immune cells (neutrophils, lymphocytes, monocytes, eosinophils, basophils) were combined into a single group. Additionally, the studies that report on selective inflammatory markers (CRP, TNF-α, IL-6, P-Selectin, CD40L, and Fibrinogen) were also combined into a single group.

#### 2.7.2. Risk of bias.

The potential risk of bias in individual studies was obtained by using the Downs and Black Checklist.^[18]^ The scores were rated as follows; excellent (25–26), good (20–24), moderate (14–19), poor (11–13), and very poor (<10). Three reviewers (NCM, AMS, and AK) were responsible for the independent judgments which were based on the 4 domains of the Black and Downs checklist tool which is reporting bias (10 items), external validity (3 items), internal validity (6 items), and selection bias (7 items). In a situation where there was a difference in opinions between NCM, AMS, and AK. PSN was then responsible for adjudication.

#### 2.7.3. Data synthesis.

For the meta-analysis of reported data, a Review Manager version 5.4 software (RevMan) Forrest plot was used.^[19]^ Using this Forrest plot, eligible data from all reported studies were analyzed depending on their sample size and the mean of the concentration of immune cells or inflammatory markers in both pre-diabetic and control groups. Additionally, the odds ratio and confidence interval were used to plot the Forrest plot where the solid lines represented the 95% confidence interval. Each reported study was represented as a horizontal line on the y-axis to list the primary author and year of study. The forest plot also included the weight of the study results at automatically detected by RevMan software.

#### 2.7.4. Sensitivity analysis.

The RevMan forest plot was also used to test for heterogeneity, where a greater overlap indicated greater homogeneity between the confidence intervals.^[19]^ Using the forest plot, *I*^2^ was then calculated where a value between 0% and 100% obtained. A value obtained less than 25% was considered to be an indication of a strong homogeneity and a value obtained greater than 75% was then considered to be an indication of a strong heterogeneity. However, a value of 50% was considered as an average value.

#### 2.7.5. Assessment of strength of evidence.

NCM, AMS, and AK will then be responsible for the assessment of the strength of evidence. The studies included in the review will then be evaluated using the Grading of Recommendations Assessment, Development and Evaluation approach (GRADE).^[20–22]^ Furthermore, the summary of finding table was then created using a GRADE pro tool.

## 3. Results

### 3.1. Search report results and eligible reports

Using the 6 databases mentioned in the search strategy paragraph, there were 4924 reports results captured. However, there were only five reports that were eligible for this systematic review and meta-analysis.

### 3.2. Scope of the reviewed reports

Among these 5 reports, two articles had information on the overall white blood cell count (WBCs).^[23–25]^ However, one article by Zang et al^[24]^ was then eliminated due to the fact that it had the hazard ratio calculated not odds ration due to the type of study conducted. Additionally, Grossmann et al^[23]^ also had additional reports on lymphocytes and monocytes. Interestingly, 4 of the eligible articles reported on inflammatory markers.^[23,25–32]^ The inflammatory markers reported were CRP, IL-6, fibrinogen and TNF-α. However, no information was obtained from markers P-selectin and soluble CD40L in the pre-diabetic state. A Microsoft Excel table was then created according to the categories mentioned in the extraction of the data paragraph. (Additional file 3). Additionally, from the reports obtained, we could extract the CRP and TNF-α in different races to investigate if there is an impact of demographic changes on CRP levels at the pre-diabetes stage. However, with the other markers, it was not possible.

### 3.3. Risk of bias assessment

All the four eligible reports undergo the risk of bias assessment using a Downs and Black checklist. The Aukour et al obtained 11 points and both Grossmann et al and Lucas et al obtained 18 points. Additionally, Sabanayagam et al obtained 19 points. However, we used all the four eligible reports since we already have less reports for the study (see Additional file 4).

### 3.4. Forrest plot report of meta-analysis and predictor of heterogeneity on immune cells and inflammatory markers articles

**Figure 1.**
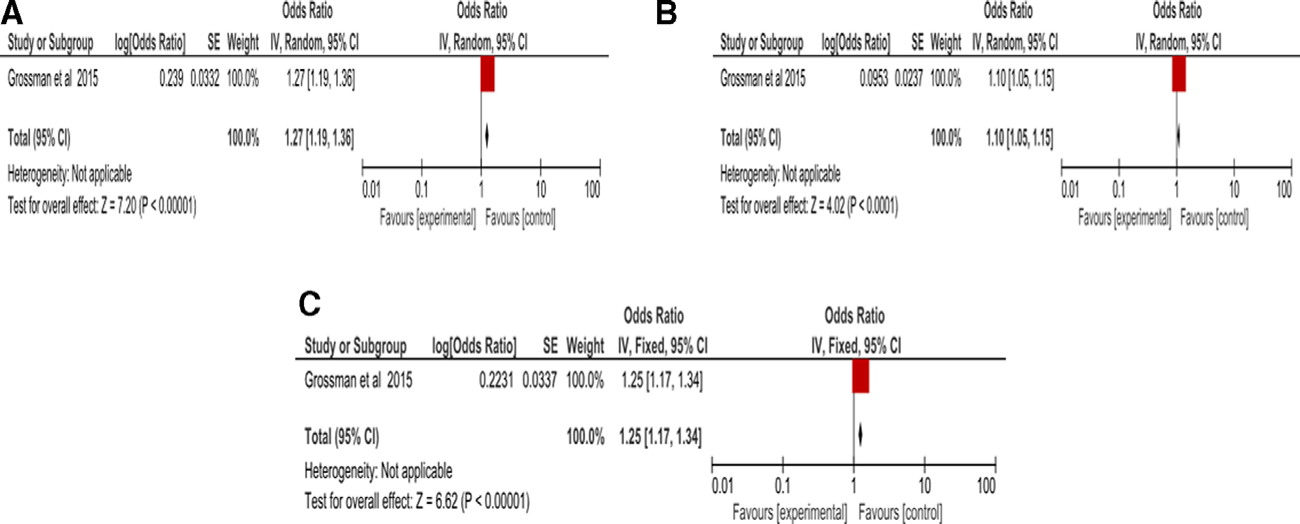
Forest plot of meta-analysis of ND vs PD in different studies where graph A represent Monocytes, B represents Lymphocytes, and C represent WBC. WBCs = white blood cell count.

**Figure 2.**
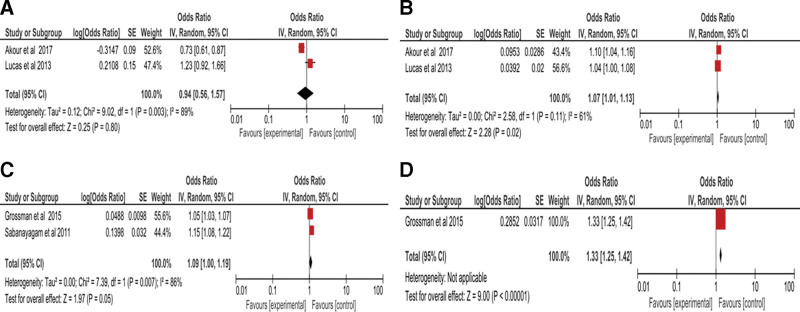
Forest plot of meta-analysis of ND vs PD in different studies where graph A represent IL-6, B represents TNF-α, C represents CRP, and D represent fibrinogen. CRP = C-reactive protein, IL-6 = Interleukin-6, TNF-α = tumour necrosis factor-alpha.

All the eligible reports were assessed for meta-bias, as shown in Figures 1 and 2. Figure 1 shows the RevMan Forrest plot of eligible evidence obtained from (A) monocytes, (B) lymphocytes, and (C) WBC. In Figure 1A, heterogeneity could not be calculated because there was only one article reporting on monocytes in pre-diabetes. However, the odds ratio obtained was 1.27 with (1.19, 1.36) CI, which favored the control (*P* < .00001). Heterogeneity could also not be calculated on Figure 1B because there was only one article reporting on lymphocytes in pre-diabetes. However, the odds ratio obtained was 1.10 with (1.05, 1.15) CI, which indicated the slight shift from control to experimental shift in pre-diabetes (*P* < .0001). Heterogeneity was not obtained from the analysis of WBC evidence collected due to one article used. However, the odds ratio of 1.22 and (1.14, 1.31) CI (*P* < .00001) was obtained, as shown in Figure 1C

The Forrest plot on Figure 2A shows a meta-analysis of IL-6 evidence of obtained indicating a strong heterogeneity of 89% at the pre-diabetes stage and pooled estimate of 0.94 (OD) and (0.56, 1.57) CL which indicates favoring the experimental group. Figure 2B shows the meta-analysis of TNF-α where I^2^ indicated a slightly above average heterogeneity of 61% with the pooled estimate of 1.07 (OD) and (1.01, 1.13) CI (*P* < .02) indicating favoring the control. Figure 2C reports on CRP meta-analysis of evidence obtained showing a strong heterogeneity of 86% with a pooled estimate of 1.09 (OD) and (1.00, 1.19) CI (*P* < .05) which favors control. Fibrinogen report obtained is indicated in the Forrest plot on Figure 2D, where an odd ratio of 1.33 and (1.25, 1.42) CI was reported at *P* < .00001.

### 3.5. Quality assessment of the pre-diabetic research reports

Table 1 shows the SoF of assessed reports with the number of studies columns, quality assessment column, effects column, quality column and importance column. These columns were then created to summarize the assessed eligible reports by the reviewers enabling them to draw conclusion to the available evidence obtained

**Table 1:** SoF of eligible assessed reports.

	n	Quality assessment				Effect	Heterogeneity %	Quality	Importance
		Indirectness	Inconsistency	Imprecision	Risk of bias	Odds ratio and confidence intervals			
Immune cells									
WBC	2	High	Moderate	Moderate	Moderate	1.22 (1.14, 1.31)	18	High	Important
Monocytes	1	High	Moderate	Moderate	Moderate	1.27 (1.19, 1.36)	n/a	Average	Important
Lymphocytes	1	High	Moderate	Moderate	Moderate	1.10 (1.05, 1.15)	n/a	Average	Important
Inflammatory markers									
IL-6	2	Moderate	High	Moderate	Moderate	0.94 (0.96, 1.57)	89	High	Important
TNF-α	2	High	High	Moderate	Moderate	1.07 (1.01, 1.13)	61	High	Important
CRP	2	High	High	Moderate	Moderate	1.09 (1.00, 1.19)	86	High	Important
Fibrinogen	1	High	High	Moderate	Moderate	1.33 (1.25, 1.42)	n/a	Average	Important

CRP = C-reactive protein, IL-6 = interleukin-6, SoF = summary of findings, TNF-α = tumour necrosis factor-alpha, WBCs = white blood cell count.

## 4. Discussion

The search of eligible data or reports in relation to the study based on immune cells concentration and selective inflammatory markers in the pre-diabetic state was very challenging due to a small amount of data available on the research of interest. Additionally, prediabetes is generally asymptomatic thus limiting the number of people that get diagnosed and thereby leading to few studies being conducted on the condition. Several studies have shown that a number of abnormalities previously ascribed only to T2DM actually begin during prediabetes.^[2,13,33]^ These abnormalities include low-grade inflammation and innate immune system suppression.^[34–37]^ However, 5 of the studies from the search obtained, were eligible for meta-analysis of this review displaying different characteristics from the criteria mentioned above.^[23,24,27,29,32]^ However, in these 5 articles one article required request on information from the authors since the report was on hazard ratio instead of odds ratio.^[24]^ The authors then indicated that the study was prospective research which then required them to use the survival analysis and cox regression analysis to estimate the hazard ratios.^[24]^ This issue then resulted in elimination of the article, as odd ratios are not applicable for the study.^[24]^ The selection of these eligible reports was also due to reviewers prioritizing the diagnostic criteria and eligibility criteria with more challenge being the sample size, age, gender and race not being in line with the required criteria in some studies for them to be meta-analyzed in this review. As an example, research by Lucas and his coworkers consists of 41 subjects which were all females and also the age ranged from 18 to 45 years which was less than the sample size indicated in our criteria and the age gap ranged from below 25 years.^[32]^ However, a study by Lucas et al was of value as it enabled us to meta-analyze the reports on CRP and calculate heterogeneity which indicated a strong heterogeneity with *I*^2^ reported to be 86%.

Interestingly, much data was collected from Grossman and his coworkers, as it reported data on WBC, monocytes, and lymphocytes.^[23]^ As much as we could not obtain the results of heterogeneity of WBC, lymphocytes and monocytes due to these results being the only single data eligible, we could however conclude that the lymphocytes favored the experimental group. Additionally, WBC and monocytes favored the control.

As for the basophils, eosinophils and neutrophils, no data was obtained and eligible for meta-analysis which is raising a strong and outstanding value of our study of interest to report on these three immune cells during pre-diabetes and in subjects aged from 25 to 45 years. The Forrest plot on IL-6 indicated the strong heterogeneity where the pooled estimate favoured the experimental group as indicated using two reports obtained.^[27,32]^ This can be hypothesized that it is possible due to the research obtained from T2D, having elevated levels of IL-6.^[38]^ Additionally, the meta-analysis for TNF-α using the obtained eligible reports indicated a slightly above average heterogeneity without much shift of the pooled estimate. This does not give us much of a conclusion because the sample size of the two eligible studies was not large enough to give accurate results but indicating a slight shift of change in concentration at pre-diabetic stage. CRP reports meta-analysis indicated a strong heterogeneity with an assumption that the accuracy of the pooled estimate is influenced by the large sample size reported by both eligible articles indicating changes during prediabetes.^[23,29]^ However, with other inflammatory markers such as CD40L and P-selectin, there was no information obtained and fibrinogen was only obtained in one article. This then raises the value of the studies that report on the previously mentioned markers to give enough clarity at the pre-diabetic stage.

From the results obtained, the evidence indicated the good quality even though it was not enough and had limitations. Additionally, there was an elimination of articles that indicated a high risk of bias, more errors on sample selection and contacted authors that did not respond. This review raises a strong value of presenting an understandable research publication that is clear and states all information such as diagnostic criteria, indicating if the participants signed informed consent and indicating the disadvantages such as loss of participants during the study or contact during follow up to avoid bias.

### 4.1. Strengths

Unavailability of enough reports to analyze for this review give strength to the value of the research of interest for us to explore more on the pre-diabetic stage at the area and country of interest. Additionally, it gives a platform to publish enough information on the immune cells and inflammatory markers that are not yet available on published work on the prediabetic state.

### 4.2. Limitations

Few articles obtained based on immune cells and inflammatory markers of interest at the prediabetic stage. Some of the articles contained small sample size; some did not specify gender and race. These characteristics limited the ability to select outstanding work based on what has been done. Additionally, less research has been done in other countries based on research concerning immune cells and inflammation at the pre-diabetic stage. Some articles do not use the ADA criteria for the diagnosis of T2D.

## 5. Conclusion

The collected evidence gives clarity that there are changes in WBCs concentration and inflammatory markers such as IL-6, TNF-α, and CRP. However, not enough evidence reported if there are changes in immune cells (monocytes, lymphocytes, basophils, eosinophils, and neutrophils) individually and markers CD40L and P-selectin at the pre-diabetes stage. The fact that we could only identify one eligible evidence on monocyte, lymphocyte, and fibrinogen, allows evaluating the changes that may be possible to occur during pre-diabetes in our research of interest. Additionally, due to the reason that some articles did not specify race and gender, we could not collect evidence if there are changes during prediabetes, which also adds value to our proposed research based on the impact of demographic changes on immunity and pre-diabetes.

## Acknowledgments

The authors would like to express gratitude to National Research Foundation (SA) for funding.

## Author contributions

NCM, AMS, and AK were responsible for brainstorming, designing the study, and then also drafted the systematic review. NCM, AMS, PS, and AK were responsible for reviewing the eligible study, analyzing the articles obtained and final draft of the manuscript. Funders had no role in development of the systematic review.

**Conceptualization:** Nomusa Christina Mzimela, Andile Khathi.

**Data curation:** Aubrey Mbulelo Sosibo, Phikelelani Siphosethu Ngubane, Andile Khathi.

**Formal analysis:** Nomusa Christina Mzimela, Aubrey Mbulelo Sosibo, Phikelelani Siphosethu Ngubane, Andile Khathi.

**Funding acquisition:** Andile Khathi.

**Methodology:** Nomusa Christina Mzimela, Andile Khathi.

**Supervision:** Andile Khathi.

**Validation:** Aubrey Mbulelo Sosibo, Phikelelani Siphosethu Ngubane, Andile Khathi.

**Visualization:** Andile Khathi.

**Writing – original draft:** Nomusa Christina Mzimela.

**Writing – review & editing:** Nomusa Christina Mzimela, Aubrey Mbulelo Sosibo, Phikelelani Siphosethu Ngubane, Andile Khathi.

## Supplementary Material
