# Supplementary material for: The changes that occur in the immune system during immune activation in pre-diabetic patients of all ethnicities, from the age of 25- to 45-years: A systematic review and meta-analysis
Source: Medicine (Baltimore). 2022 Dec 23;101(51):e30903. doi: 10.1097/MD.0000000000030903 (PMC9794255; doi:10.1097/MD.0000000000030903)
Supplement: Supplementary file 2 [file medi-101-e30903-s002.pdf]

PRISMA 2009 Flow Diagram

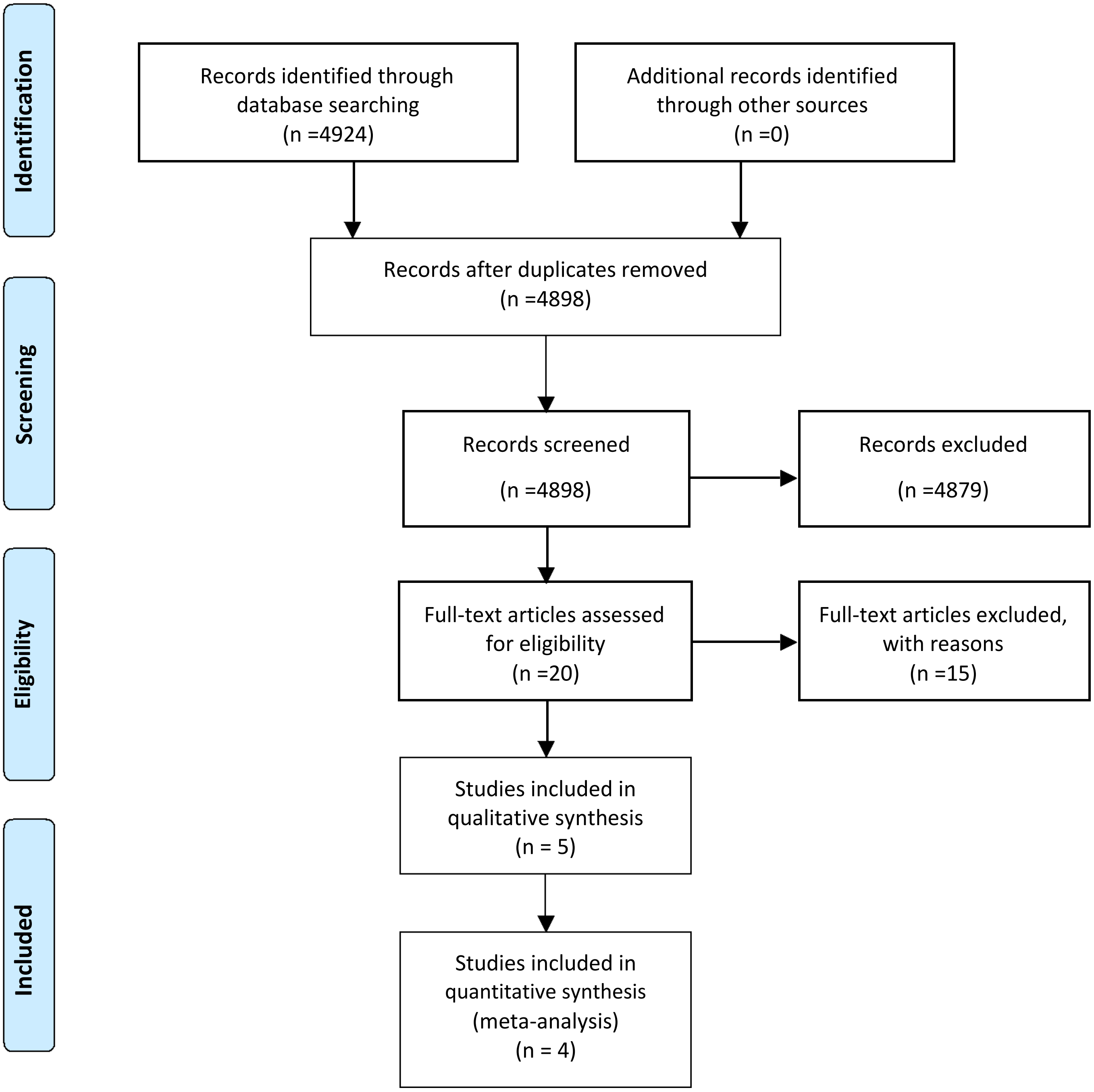

From: Moher D, Liberati A, Tetzlaff J, Altman DG, The PRISMA Group (2009). *P*referred *R*eporting *I*tems for *S*ystematic Reviews and *M*eta-*A*nalyses: The PRISMA Statement. PLoS Med 6(7): e1000097. doi:10.1371/journal.pmed1000097

For more information, visit www.prisma-statement.org.
